# Magnetic resonance imaging features of massive ovarian edema in pregnancy: utility for decisions in expectant management

**DOI:** 10.1186/s40064-016-3123-3

**Published:** 2016-08-30

**Authors:** Aiko Gobara, Takeshi Yoshizako, Rika Yoshida, Naruhito Okada, Ken Makihara, Hajime Kitagaki

**Affiliations:** 1 Department of Radiology, Shimane University Faculty of Medicine, P.O. Box 00693-8501, 89-1 Enyacho, Izumo, Japan; 2 Department of Radiology, Ohda Municipal Hospital, P.O. Box 00694-0063, 1428-3 Ohdacho, Ohda, Japan; 3 Department of Gynecology, Ohda Municipal Hospital, P.O. Box 00694-0063, 1428-3 Ohdacho, Ohda, Japan

**Keywords:** Magnetic resonance imaging, Massive ovarian edema, Pregnancy

## Abstract

**Introduction:**

Massive ovarian edema (MOE) is a rare disease and few reports have described the magnetic resonance (MR) imaging manifestations in pregnancy.

**Case description:**

We report here a case of MOE in a patient at 12 weeks’ gestation. Abdominal T2-weighted MR images showed asymmetric ovarian enlargement in a teardrop configuration, hyperintense peripherally displaced follicles, and twisting of the vascular pedicle between the enlarged ovary and uterus. The diagnosis of MOE due to ovarian torsion was confirmed by exploratory laparotomy. Preoperative imaging, especially the MR imaging could distinguish MOE from other conditions and demonstrate the relations of adjunct organ, and allowed for untwisting during laparotomy with successful preservation of the ovary.

**Discussion and evaluation:**

Ultrasonography is important in detecting, evaluating, and determining the malignant potential of adnexal masses in pregnancy, but its findings may be nonspecific and then MR may assist characterization. This case was tentatively diagnosed as typical MOE by preoperative imaging, but the shape and location of the hugely enlarged ovarian mass suggested torsion of the ovarian pedicle. In our case, the diagnosis was confirmed by exploratory laparotomy and the pedicle was successfully untwisted.

**Conclusion:**

MR imaging proved useful for decisions on expectant management of MOE in pregnancy, and the patient’s affected ovary could be preserved.

## Background

 Massive ovarian edema (MOE) is a tumor-like enlargement of the ovary characterized by an accumulation of edema fluid within the ovarian stroma. It mainly resulted from partial or intermittent torsion of the ovary with obstruction of venous and lymphatic drainage. MOE is a rare disease and few reports have demonstrated magnetic resonance (MR) findings of MOE in pregnancy (Kalstone et al. 1969; Geist et al. 2005; Hall et al. 1993; Gustafson et al. 1954; Chervenak et al. 1980; Weinreb et al. 1986; Lambert and Lessard 1987; Schmidt et al. 2007; Coakley et al. 2010). We report here a case of MOE in a patient at 12 weeks’ gestation. Preoperative imaging, especially the MR imaging could distinguish MOE from other conditions and understand the relations of adjunct organ, and allowed for untwisting during laparotomy with successful preservation of the ovary.

## Case report

**Fig. 1:**
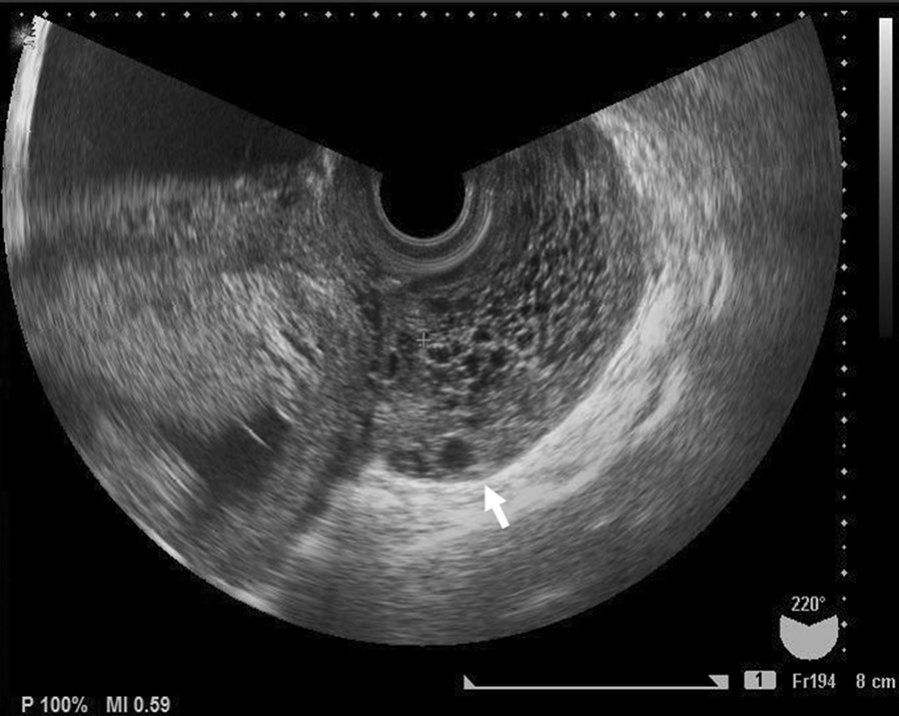
Transvaginal ultrasonography shows a solid mass (*arrow*) with hypoechogenic cysts in the Douglas’ pouch

**Fig. 2:**
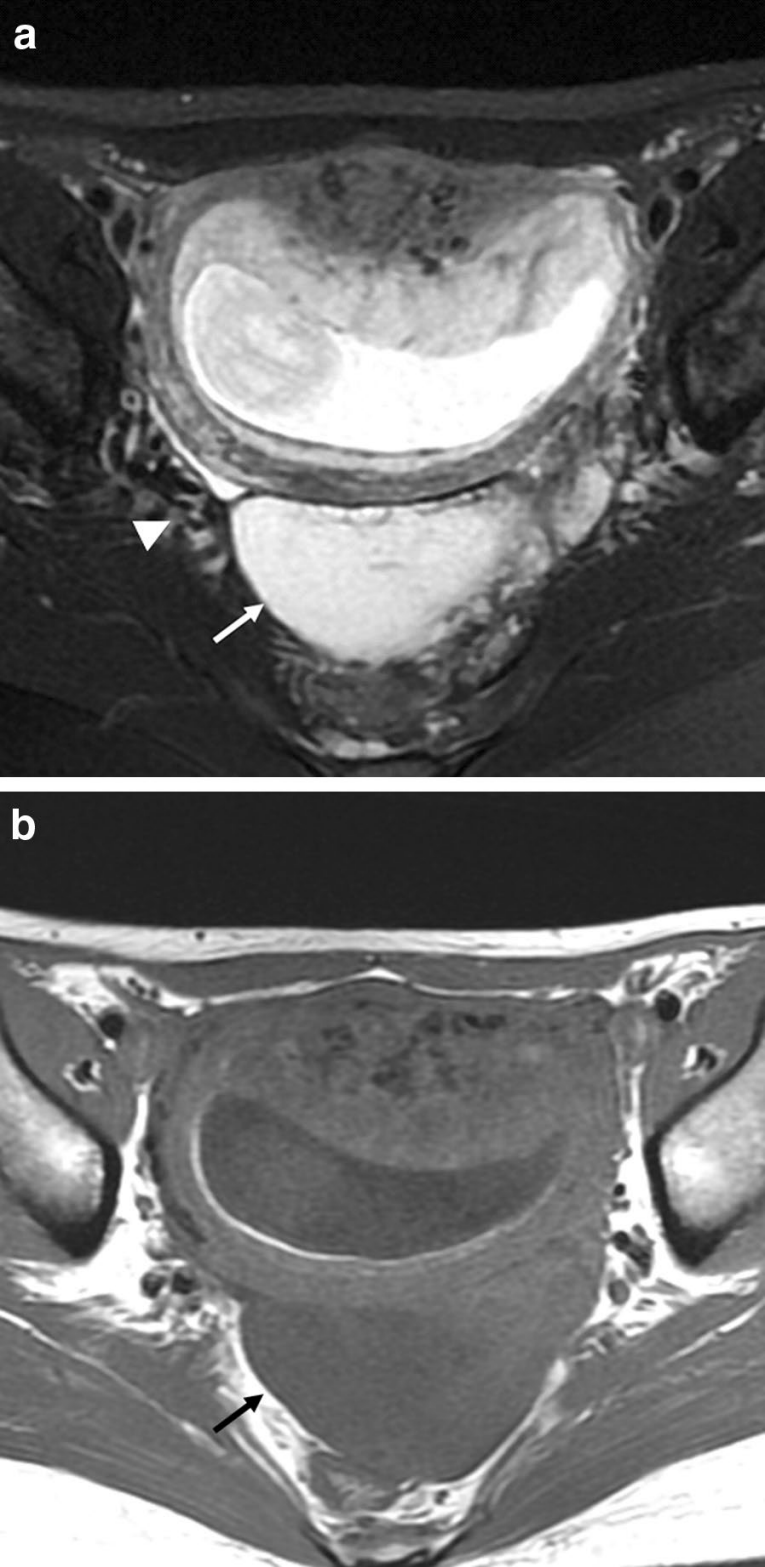
**a** Fat-suppressed T2-weighted image shows the markedly high intensity mass (*arrow*) with peripherally displaced follicles. The ovary has a teardrop configuration and the vascular pedicle appears twisted (*arrowhead*) beside the enlarged ovary and uterus. **b** T1-weighted image shows a homogenous low-intensity mass (*arrowhead*)

A 24-year-old woman presented with lower abdominal pain at 12 week’ gestation. Abdominal and bimanual pelvic examination revealed a relatively immovable, goose-egg-sized, solid mass in the left region of the uterine adnexa. On the transvaginal ultrasound, a solid mass (68 × 36 × 67 mm) with multiple small follicles was located in Douglas’ pouch (Fig. 1). Abdominal pain gradually worsened, and by 14 weeks’ gestation the mass had grown to 99 × 61 × 60 mm. The mass was presumed as MOE or fibro-thecoma, but not able to deny malignant tumor. Pelvic MR imaging was performed on a 1.5-T whole-body MR scanner (Signa; General Electric Medical Systems, Milwaukee, Wis), with patients in the supine position using a pelvic surface coil (General Electric Medical Systems, Milwaukee, Wis). MR imaging revealed a defined mass in Douglas’ pouch, which was presumed to be the left ovary. The mass appeared as a high-intensity region with peripherally displaced follicles on fat-suppressed T2-weighted MR images (Fig. 2a) and as a homogenous low-intensity region on T1-weighted images (Fig. 2b). The ovary had a teardrop configuration and the vascular pedicle appeared twisted beside the enlarged ovary and uterus (Fig. 2a). The right ovary appeared normal. Tumor markers (CEA, CA125) were not elevated. Based on these imaging findings and laboratory results, we made a preoperative presumed diagnosis of MOE due to ovarian torsion. The abdominal pain continued and the mass grew bigger, therefore, exploratory laparotomy was performed 2 days later.

**Fig. 3:**
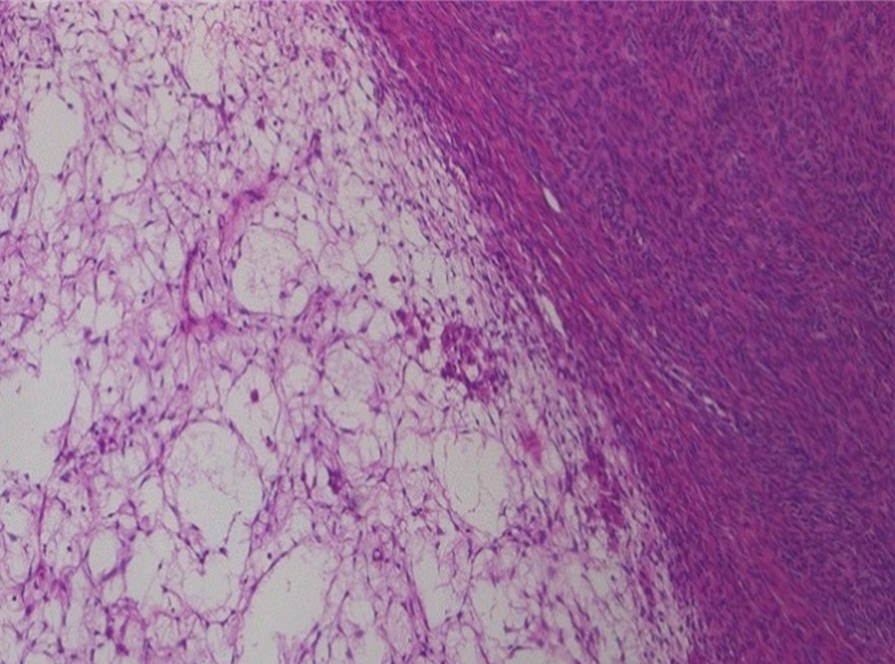
The ovary shows edematous change of the stroma with low cellularity (hematoxylin and eosin, ×100)

The vascular pedicle extending to the left ovary was rotated 360° and it was successfully untwisted. The left ovary had edematous components. No hemorrhage or necrosis was found. Macroscopically, the right ovary had no abnormal findings. Partial resection of the enlarged left ovary was done and the ovary was fixed to prevent future twisting. Histologically, an edematous and hypo-cellular ovarian stroma, together with preserved ovarian architecture (Fig. 3) with final diagnosis was MOE. Course of the pregnancy was uneventful.

## Discussion

MOE is a rare condition presenting itself with enlargement of the ovary due to gross diffuse stromal edema. The literature contains only around 130 cases since the first case was reported by Kalstone et al. (1969), and only 9 cases have been reported during pregnancy (Hall et al. 1993; Gustafson et al. 1954; Chervenak et al. 1980; Weinreb et al. 1986; Lambert and Lessard 1987; Schmidt et al. 2007; Coakley et al. 2010). While MOE may occur at any maternal age, it tends to be more common in young women, with a mean age at diagnosis of 20 years (Geist et al. 2005; Chervenak et al. 1980). The mean diameter of the swollen ovary is 10.4 cm (Patty et al. 1993). The condition may occur unilaterally in the right ovary. The most common presenting symptom is abdominal pain, accompanied in some cases by menstrual irregularity, infertility, and/or hormonal effects such as virilization. MOE is thought to result from interference with venous and lymphatic drainage due to partial or intermittent torsion of the ovary or mesovarium (Geist et al. 2005; Hall et al. 1993).

Several previous reports have discussed MOE imaging diagnosis using ultrasonography and MR imaging (Hall et al. 1993; Lee et al. 1993; Kramer et al. 1997). On ultrasound, MOE resembles a solid ovarian tumor with multiple peripheral ovarian follicles, and patients have often undergone oophorectomy based on this imaging presentation. On MR imaging, T2-weighted images show an extensively enlarged ovary with edematous stroma of high intensity accompanied by peripherally displaced follicles. T1-weighted images show the ovary as homogenous low intensity, but if congestive hemorrhage is present, it may appear with slightly higher intensity. Asymmetry and stromal T2 hyper-intensity of the ovary may help distinguish MOE from other pathological conditions such as polycystic ovary syndrome and neoplastic ovary.

Adnexal masses in pregnancy are found in 1–2 % of pregnancies and 1–3 % of these will be malignant (Chiang and Levine 2004). Ultrasonography is important in detecting, evaluating, and determining the malignant potential of these masses, but its findings may be nonspecific and therefore MR may assist characterization. Most studies report MR during pregnancy to be safe, but several animal studies have indicated the possibility of teratogenic effects in early pregnancy (Coakley et al. 2004). Although these studies may not be applicable to humans, a more cautious approach should be taken when MR is required during the first trimester. In our case, the findings of ultrasonography were inconclusive and insufficient to decide the treatment strategy. Accordingly MR was considered as a useful adjunct.

MOE in pregnancy can result from chronic vascular congestion of the ovary, with the ovarian pedicle either in a state of torsion or trapped between the gravid uterus and an adjacent fixed anatomic structure such as the pelvic inlet or abdominal wall. In our case, the left ovarian pedicle turned on itself 360 degrees, explaining the teardrop configuration of the ovary. Coakley et al. (2010) reported on spontaneous resolution with good pregnancy outcome.

There is currently no standard treatment for MOE. It is generally accepted that the ovary should be preserved in young women.

The present case was tentatively diagnosed as typical MOE by preoperative imaging, but the shape and typical appearance of the enlarged ovary suggested torsion of the ovarian pedicle. This was confirmed and rectified during exploratory laparotomy. Imaging diagnosis can be helpful for deciding patient management. It has been reported that MOE in pregnancy can be managed expectantly.

## Conclusion

MR imaging proved to be useful in the management of a case of MOE in pregnancy.

## Abbreviations

MOE: massive ovarian edema
MR: magnetic resonance
